# A codesigned integrated kidney and diabetes model of care improves patient activation among patients from culturally and linguistically diverse backgrounds

**DOI:** 10.1111/hex.13859

**Published:** 2023-08-27

**Authors:** Edward Zimbudzi, Clement Lo, Sanjeeva Ranasinha, Tim Usherwood, Kevan R. Polkinghorne, Gregory Fulcher, Martin Gallagher, Stephen Jan, Alan Cass, Rowan Walker, Grant Russell, Greg Johnson, Peter G. Kerr, Sophia Zoungas

**Affiliations:** ^1^ School of Public Health and Preventive Medicine Monash University Melbourne Victoria Australia; ^2^ Monash Nursing and Midwifery Monash University Melbourne Victoria Australia; ^3^ Department of Nephrology Monash Health Melbourne Victoria Australia; ^4^ Diabetes and Vascular Medicine Unit, Monash Health Melbourne Victoria Australia; ^5^ The George Institute for Global Health University of New South Wales Sydney New South Wales Australia; ^6^ Department of General Practice, Sydney Medical School University of Sydney Sydney New South Wales Australia; ^7^ School of Clinical Sciences Monash University Melbourne Victoria Australia; ^8^ Department of Diabetes, Endocrinology and Metabolism, Royal North Shore Hospital University of Sydney Sydney New South Wales Australia; ^9^ Northern Clinical School University of Sydney Sydney New South Wales Australia; ^10^ Concord Clinical School University of Sydney Sydney New South Wales Australia; ^11^ Sydney Medical School University of Sydney Sydney New South Wales Australia; ^12^ Menzies School of Health Research Charles Darwin University Casuarina Northern Territory Australia; ^13^ Department of Renal Medicine Alfred Health Melbourne Victoria Australia; ^14^ School of Primary Health Care Monash University Melbourne Victoria Australia; ^15^ Diabetes Australia Canberra Australian Capital Territory Australia

**Keywords:** chronic kidney disease, culturally and linguistically diverse background, diabetes, integrated kidney and diabetes model of care, patient activation

## Abstract

**Background:**

Little is known about the relationship between patients' cultural and linguistic backgrounds and patient activation, especially in people with diabetes and chronic kidney disease (CKD). We examined the association between culturally and linguistically diverse (CALD) background and patient activation and evaluated the impact of a codesigned integrated kidney and diabetes model of care on patient activation by CALD status in people with diabetes and CKD.

**Methods:**

This longitudinal study recruited adults with diabetes and CKD (Stage 3a or worse) who attended a new diabetes and kidney disease service at a tertiary hospital. All completed the patient activation measure at baseline and after 12 months and had demographic and clinical data collected. Patients from CALD backgrounds included individuals who spoke a language other than English at home, while those from non‐CALD backgrounds spoke English only as their primary language. Paired *t*‐tests compared baseline and 12‐month patient activation scores by CALD status.

**Results:**

Patients from CALD backgrounds had lower activation scores (52.1 ± 17.6) compared to those from non‐CALD backgrounds (58.5 ± 14.6) at baseline. Within‐group comparisons showed that patient activation scores for patients from CALD backgrounds significantly improved by 7 points from baseline to 12 months follow‐up (52.1 ± 17.6–59.4 ± 14.7), and no significant change was observed for those from non‐CALD backgrounds (58.5 ± 14.6–58.8 ± 13.6).

**Conclusions:**

Among patients with diabetes and CKD, those from CALD backgrounds report worse activation scores. Interventions that support people from CALD backgrounds with comorbid diabetes and CKD, such as the integrated kidney and diabetes model of care, may address racial and ethnic disparities that exist in patient activation and thus improve clinical outcomes.

**Patient or Public Contribution:**

Patients, caregivers and national consumer advocacy organisations (Diabetes Australia and Kidney Health Australia) codesigned a new model of care in partnership with healthcare professionals and researchers. The development of the model of care was informed by focus groups of patients and healthcare professionals and semi‐structured interviews of caregivers and healthcare professionals. Patients and caregivers also provided a rigorous evaluation of the new model of care, highlighting its strengths and weaknesses.

## 1 INTRODUCTION

Patient activation, defined as an individual's knowledge, skills and confidence in managing chronic diseases,^1^ is associated with important health outcomes. Demographic characteristics such as older age,^2^, ^3^, ^4^ low education,^2^, ^5^ low socioeconomic status^3^, ^5^ and smoking^6^ are associated with low activation. Individuals with lower levels of activation are more likely to be obese^7^ and less likely to achieve cholesterol and glycated haemoglobin targets.^8^ With respect to healthcare utilisation, individuals who report lower activation levels are more likely to be hospitalised,^9^ have a longer length of stay in hospital^10^ and have greater healthcare costs.^11^

In individuals experiencing multimorbidity, especially those with long‐term health conditions like depression, type 2 diabetes mellitus, dementia and frailty,^12^ activation levels are low. Similarly, activation levels are low among patients with comorbid diabetes and chronic kidney disease (CKD), especially those who have advanced kidney disease, are older and have poor self‐reported health status.^13^, ^14^

While a number of factors associated with activation in patients with comorbid diabetes and CKD are now known,^13^, ^14^ there is a knowledge gap regarding the relationship between patient activation and patients' cultural and linguistic backgrounds. This gap is important, given that people from culturally and linguistically diverse (CALD) backgrounds experience a higher burden of disease and difficulties in accessing health services,^15^ which impacts significantly on their health and quality of life. Among patients from CALD backgrounds, access to health services is influenced by challenges at an individual and family level, community and organisational level and systems and policy levels. These challenges include poor health literacy, multimorbidity, diminishing healthy migrants' effect, unhealthy food behaviours and lifestyles, language and communication problems, inadequate interpretation services and inadequate health systems and services to address the needs of CALD populations.^15^ Additionally, patients from CALD backgrounds are largely underrepresented in research, which limits evidence in how to improve access, utilisation and policies specific to their community needs.^16^

Furthermore, the patient activation measure (PAM), a tool that evaluates the patients' perceived knowledge, skills and confidence to engage in self‐management activities, has been shown to perform differently to patients of varied cultural and linguistic backgrounds.^17^ The risk of bias in the PAM increases given its initial development was conducted through testing with an 88% White population who were predominantly fluent in the English language.^1^ This highlights the importance of not only assessing variations in PAM across cultural and linguistic backgrounds but also evaluating PAM differences within groups.

Another key knowledge gap is the impact of person‐centred integrated models of care on activation levels of people with diabetes and CKD from CALD backgrounds. Person‐centred integrated models of care ensure that patients' values and concerns inform the management of long‐term conditions instead of focusing on a standard set of disease management processes designed by health professionals.^18^ This approach encourages patients to actively participate in the selection of their treatment goals and to collaborate with health professionals regarding their specific needs for treatment and support of their chronic diseases.^19^ Integrated care delivered in this way has been reported to improve patient satisfaction, perceived quality of care and access to services^20^ and to lower costs compared with usual care.^21^ We hypothesise that person‐centred integrated models of care may improve activation levels for patients from CALD backgrounds due to several reasons. First, person‐centred integrated models of care provide an opportunity for patients to build a certain level of trust in healthcare workers and the healthcare system, allowing for sharing of ideas and decision‐making, which is important for improving patient activation.^22^ Second, given that most individuals from CALD backgrounds are disproportionately impacted by discrimination within the healthcare system,^23^ asking them to embrace interventions that promote activation may improve their experience of the healthcare system. An alternative approach is care provided through person‐centred care models that focus on addressing implicit attitudes and behaviours among clinicians and the healthcare systems, which have been shown to negatively influence healthcare professionals' willingness to engage in patient‐centred care, provide referrals to specialised treatment or even adhere to evidence‐based guidelines when serving diverse populations.^24^, ^25^

The purpose of this study was not only to examine the association between patient activation and CALD background in patients with diabetes and CKD but also to determine the impact of a codesigned integrated kidney and diabetes model of care on patient activation for patients from CALD backgrounds.

## 2 METHODS

### 2.1 Study design, setting and participants

Adult patients (over 18 years) with diabetes and CKD who were receiving care from the Diabetes Kidney Service (DKS)^26^ at Monash Health between January 2015 and August 2017 were recruited and followed up for 12 months. The study design, recruitment and follow‐up of participants have been previously described in detail.^27^, ^28^ In brief, participants with a diagnosis of diabetes (either type 1 or type 2) and CKD stages 3–5 (estimated glomerular filtration rate [eGFR] < 60 mL/min/1.73 m^2^), including dialysis, were included in the study. This study focused on people with more advanced CKD and diabetes, which was the group we deemed had the greatest need. The diagnosis of diabetes was written in medical records and/or confirmed by the laboratory. Patients were considered to have CKD if they had a sustained eGFR < 60 mL/min/1.73 m^2^ (i.e., two or more eGFR readings <60 mL/min/1.73 m^2^ over a 3‐month period) calculated using the CKD‐EPI (Chronic Kidney Disease Epidemiology Collaboration) formula.^29^ Key exclusion criteria were an eGFR ≥ 60 mL/min/1.73 m^2^ and a functioning kidney transplant. The study was conducted by following the Strengthening the Reporting of Observational Studies in Epidemiology recommendations.^30^ We obtained ethics approval from Monash University and Monash Health Human Ethics Review Committees.

### 2.2 The diabetes and kidney disease model of care

We have previously described the diabetes and kidney disease model of care in detail.^28^ In brief, this model of care was codesigned by healthcare professionals (general practitioners, endocrinologists, nephrologists and nurse practitioners), patients with diabetes and CKD and patient advocacy groups such as Diabetes Australia and Kidney Health Australia in 2015. Findings from a large multisite formative evaluation of the barriers and enablers of current health services for diabetes and CKD and the needs of patients, carers and their health professionals informed the design of this model of care.^31^ This study highlighted the presence of guideline‐care gaps among patients with diabetes and CKD as well as barriers to health care that included poor continuity of care, inadequate understanding/education about CKD, feeling unwell, inadequate support from family and friends, conflicting advice from specialists and poor communication among specialists, and these varied across CKD stage and hospitals.^31^

The diabetes and kidney disease model of care aims to provide patient‐centred, coordinated multidisciplinary assessment and management of patients with comorbid diabetes and CKD in partnership with primary care. The service focuses on improving patient self‐management, communication and better coordination of care between health professionals. General practitioners remain the coordinators of patient care within a team including an endocrinologist and nephrologist, specialist registrars in endocrinology and nephrology, diabetes and renal nurse practitioners, dietitian, administration and a research officer (for evaluation and continual improvement of the service). Patient self‐management is achieved through motivational interviewing and tailored education. Patients also get a diabetes‐kidney care plan after each visit to ensure that they are updated with their care goals/management. The service utilises professional interpreting services in preference to family members for non‐English speaking patients. Referrals to the integrated service included patients with type 1 or type 2 diabetes with an eGFR < 60 mL/min/1.73 m^2^.

## 3 STUDY MEASURES

### 3.1 Demographic and clinical variables

Age, gender, CALD status, stage of kidney disease and duration of diabetes were obtained from a questionnaire (Supporting Information: Appendix S1), which was prospectively completed by site study staff or the clinician, using standardised procedures from the doctor's notes and laboratory results from the clinic.

### 3.2 CALD background

The definition of CALD background has evolved from the one proposed by the Australian Bureau of Statistics that utilised the country of birth, language spoken at home, English proficiency or other characteristics (including year of arrival in Australia) to determine CALD status.^32^ The most recent literature suggests that CALD status would best be defined as people born in non‐English speaking countries and/or who do not speak English at home.^33^ For the purpose of our study, we used the latter definition mainly because we collected data on country of birth as well as the primary language spoken at home. We used the term CALD over ‘Non‐English‐Speaking Background’ for it is inclusive of individuals based on more than simply language and more welcoming and reflective of the diversity of the entire population.^34^

### 3.3 Patient activation

We evaluated patients' level of involvement in their health care by administering the PAM‐13 at baseline and after 12 months.^35^ This 13‐item measure has a similar reliability and validity to the 22‐item version across different ages, genders and health condition status (Cronbach's *α* of .91 and a Rasch person statistic of 0.81 for the real and 0.85 for the model on which it was based).^1^ The PAM scale has 13 questions, with four alternative responses to each of the 13 items, namely, ‘disagree strongly, disagree, agree and agree strongly’, and the fifth response option, ‘not applicable’ (N/A), was available for all items.^35^ A standardised spreadsheet provided by Insignia Health® was used to calculate the PAM score.^36^ Participants who responded to less than seven items or who answered all questions with ‘disagree strongly’ or ‘agree strongly’ were excluded as per the developer's recommendations. The mean PAM score was then calculated on all items leaving out the ones thought to be nonapplicable by the participants. The raw mean score was converted into a standardised activation score ranging from 0 to 100, creating the PAM scores which were classified into the four levels of activation: Level 1 (score: <47.0), Level 2 (score: 47.1–55.1), Level 3 (score: 55.2–67.0) and Level 4 (score: >67.0) as per Insignia Health® scoring rules.^36^

### 3.4 Statistical analysis

Participants who completed the PAM questionnaire were included in this analysis. First, the *χ* ^2^ test or the independent *t*‐test examined differences in covariates by CALD status at baseline. Second, using the PAM score as a continuous variable, a multivariate regression analysis was performed to assess the relationship between patient activation and CALD background, adjusting for factors that have been known to influence patient activation at baseline. These factors include gender, age, duration of diabetes, stage of CKD and patient‐reported barriers to health care. Given the importance of these covariates to patient activation, they were all included in the model. The process of determining patient‐reported barriers to health care among patients with comorbid diabetes and CKD has been reported previously.^37^ Third, paired *t*‐tests examined changes in patient activation within groups. A sensitivity analysis compared baseline activation scores for patients from CALD background who did not complete 12 months follow‐up and those who did to determine the effect of baseline patient activation scores on participation and retention of these patients in the study. Confidence intervals (CIs) were reported at the 95% level and results were considered significant at conventional *p* < .05 level. All analyses were performed using Stata version 16.0 (StataCorp).

## 4 RESULTS

### 4.1 Participants

**Figure 1:**
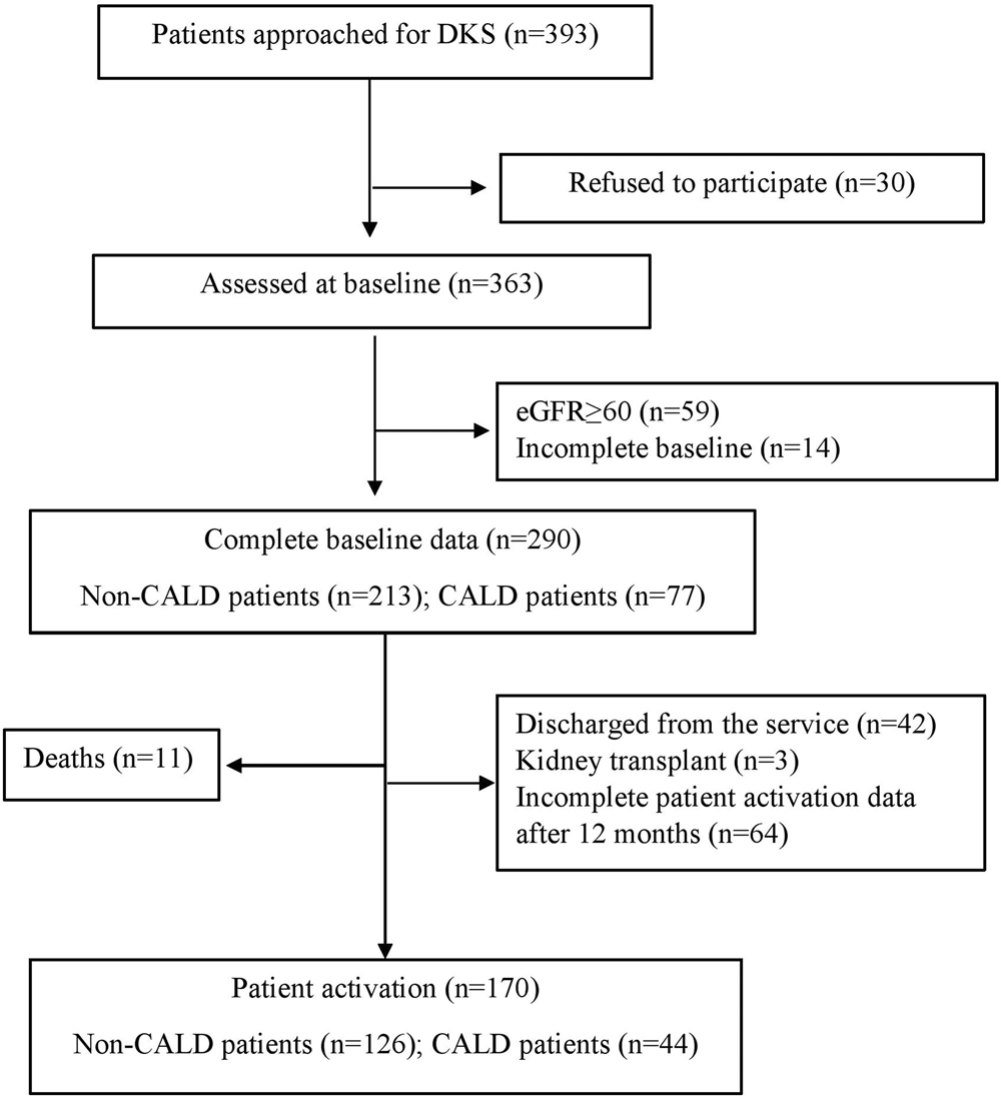
Strengthening the Reporting of Observational Studies in Epidemiology flow diagram of included patients. CALD, culturally and linguistically diverse; DKS, diabetes kidney service; eGFR, estimated glomerular filtration rate.

Of a total of 393 patients screened, 290 met the inclusion criteria for the model of care evaluation (Figure 1). Of these, 77 (27%) were from CALD backgrounds. During follow‐up, 11 died (3.8%) before the 12‐month visit and 170 (57%) completed the 12‐month patient activation questionnaires. The baseline demographic and clinical characteristics of the study population are shown in Table 1. At baseline, the mean (SD) age for all patients was 67 ± 12 years, with a predominance of men (64%), and patients were evenly distributed across all stages of kidney disease. There were no differences in age, gender, stage of kidney disease, duration of diabetes, body mass index, barriers to health care and access to diabetes and kidney care by CALD status (all *p* > .05).

**Table 1:** Patient characteristics by CALD status at baseline.

	All patients (*N*/%)	Non‐CALD background (*N*/%)	CALD background (*N*/%)	*p*‐Values
Participants (*n*)	290	213 (73.4)	77 (26.6)	
Age (years, SD)	66.6 (11.9)	66 (11.6)	68.5 (12.2)	.13
Gender				
Female	106 (36.5)	76 (35.9)	30 (39.5)	
Male	184 (63.5)	136 (64.1)	46 (60.5)	.32
Duration of diabetes (years)	16.1 (8.9)	16.2 (9.0)	16 (9.0)	.91
Stages of CKD				
3a	56 (19.3)	38 (17.9)	18 (23.7)	
3b	109 (37.5)	83 (39.2)	26 (34.2)	
4	68 (23.5)	48 (22.6)	20 (26.3)	
5 (Including dialysis)	57 (19.7)	43 (20.3)	12 (15.8)	.53
Body mass index	34.4 (10.5)	35.6 (11.5)	30.7 (5.2)	.10
Barriers to health care				
Yes	146 (50.3)	104 (49.1)	40 (52.6)	
No	144 (49.7)	108 (50.9)	36 (47.4)	.59
Difficult to access diabetes care				
Yes	48 (16.7)	34 (16.3)	13 (17.1)	
No	289 (83.3)	175 (83.7)	63 (82.9)	.87
Difficult to access kidney care				
Yes	47 (17.6)	35 (17.9)	12 (17.4)	
No	220 (82.4)	161 (82.1)	57 (82.6)	.93
Patient activation (mean, SD)	56.8 (15.6)	58.5 (14.6)	52.1 (17.6)	.002

Abbreviations: CALD, culturally and linguistically diverse background; CKD, chronic kidney disease.

### 4.2 Patient activation

**Figure 2:**
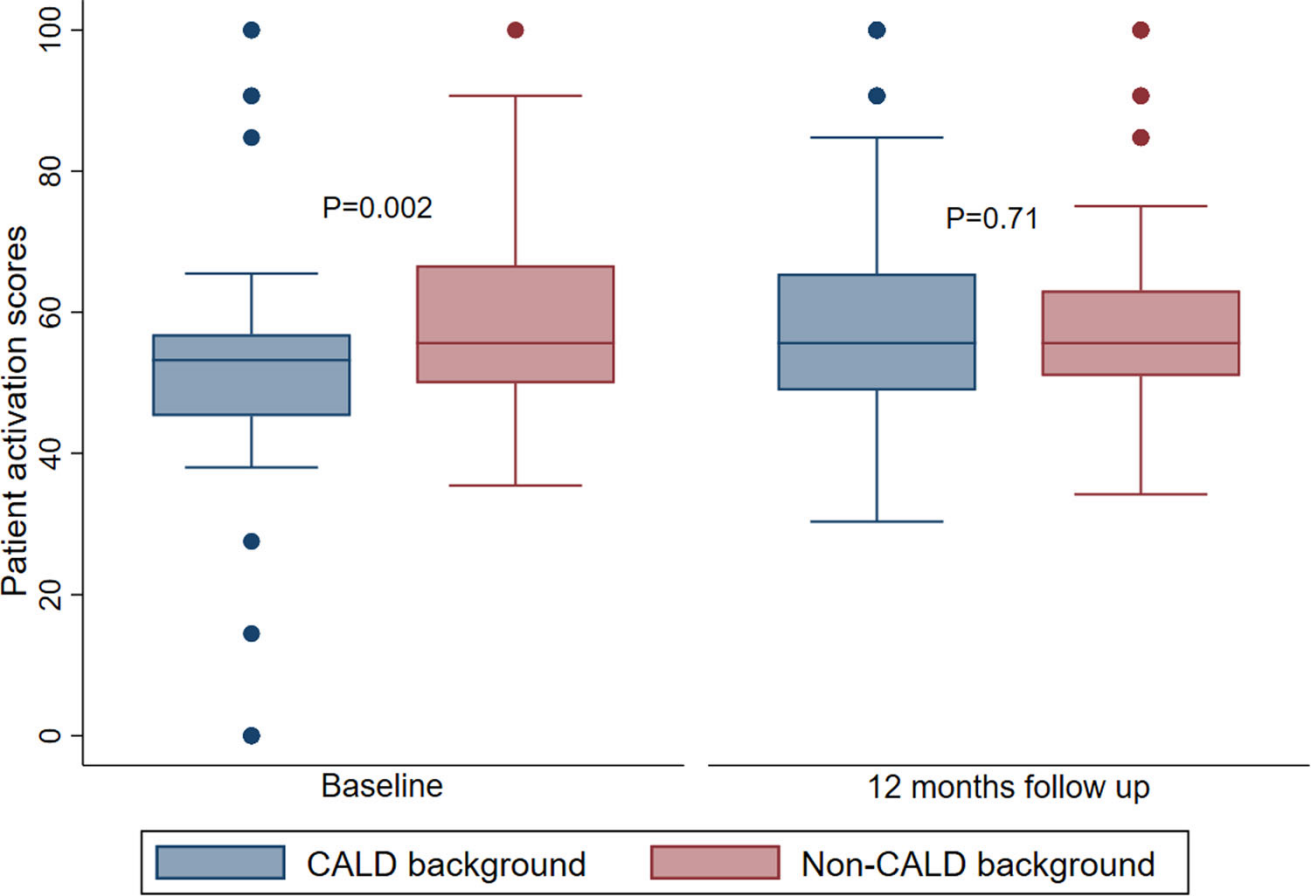
Patient activation scores for patients from CALD and non‐CALD backgrounds at baseline and 12 months follow‐up. CALD, culturally and linguistically diverse background.

**Figure 3:**
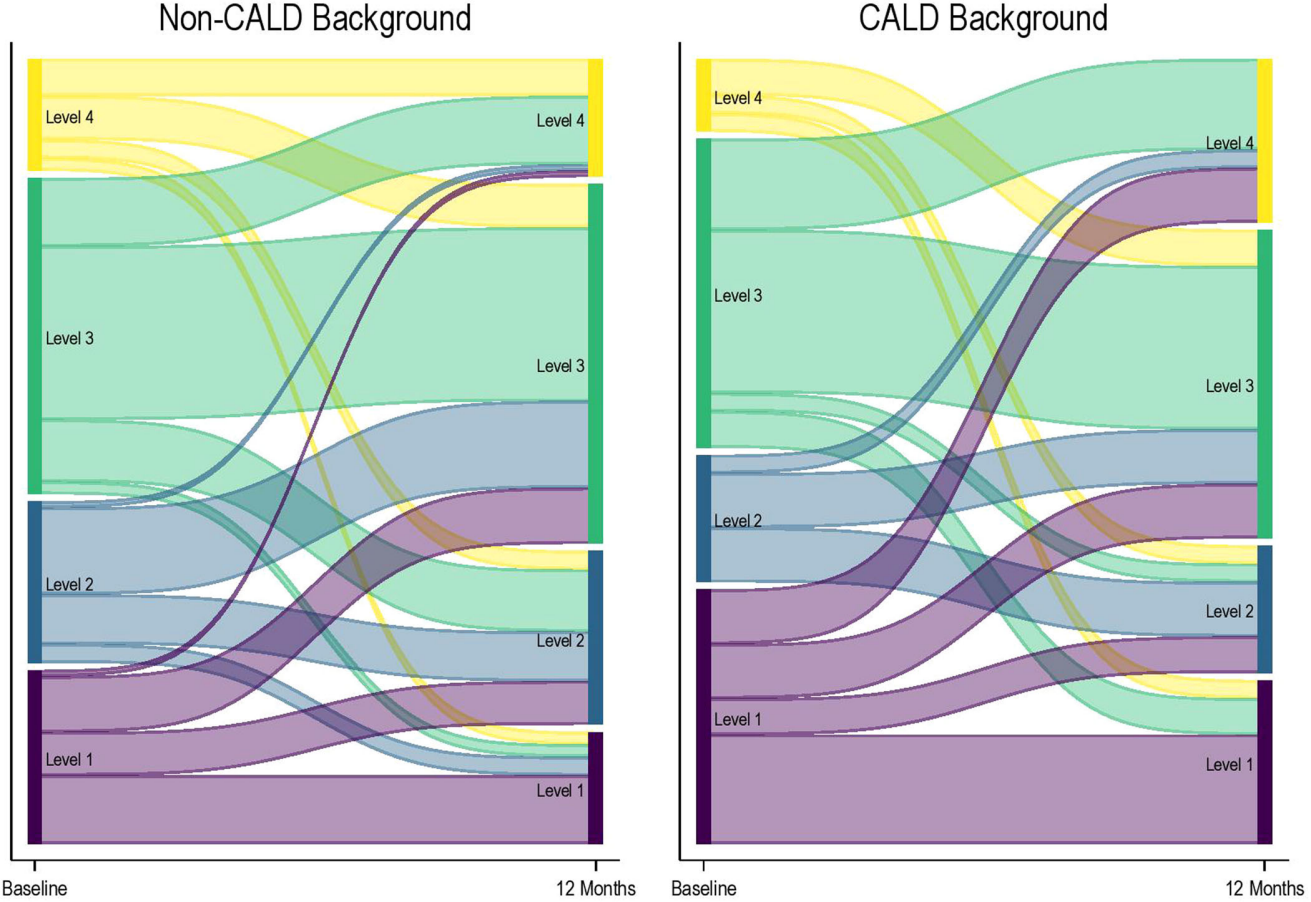
Sankey plot showing changes in patient activation levels from baseline to 12 months follow‐up. Level 1 (score: <47.0), Level 2 (score: 47.1–55.1), Level 3 (score: 55.2–67.0) and Level 4 (score: >67.0). CALD, culturally and linguistically diverse background.

At baseline, individuals from CALD backgrounds had significantly lower patient activation scores (52.1 ± 17.6) compared to those from non‐CALD backgrounds (58.5 ± 14.6) (mean difference: −6.4, 95% CI: −10.8 to −2.5, *p* = .002) (Figure 2 and Table 1). Among patients with low activation (Levels 1 and 2), 40% were from non‐CALD backgrounds and 50% were from CALD backgrounds (Figure 3). In multivariate analysis adjusted for age, gender, duration of diabetes, eGFR and presence of barriers to health care at baseline, participants from CALD backgrounds scored on average 7 points lower than those from non‐CALD backgrounds (Table 2).

**Table 2:** Adjusted multivariate regression analysis of the relationship between patient activation and CALD status at baseline.

Variable	*β* Coefficient (95% CI)	*p*‐Values
CALD background (ref: non‐CALD background)	−6.8 (−11.3 to −2.3)	.003
Age (over 65 years) (ref: <65 years)	−3.1 (−7.1 to 0.8)	.12
Male (ref: female)	−1.4 (−5.5 to 2.7)	.50
Diabetes duration (years)	.2 (−0.1 to 0.4)	.18
eGFR (mL/min/1.73 m^2^)	.1 (−0.1 to 0.2)	.23
Barriers to health care (ref: no)	−8.7 (−12.6 to −4.9)	<.0001

Abbreviations: CALD, culturally and linguistically diverse background; CI, confidence interval; eGFR, estimated glomerular filtration rate.

At 12 months, no difference in activation scores was observed between individuals from non‐CALD and CALD backgrounds (Figure 2). Forty percent of patients from CALD background and non‐CALD backgrounds had low levels of activation (Figure 3). Within‐group comparisons showed that patient activation scores for patients from CALD backgrounds significantly improved by 7 points from baseline to 12 months follow‐up (52.1 ± 17.6–59.4 ± 14.7), and no significant change was observed for those from non‐CALD backgrounds (58.5 ± 14.6–58.8 ± 13.6) (Figure 2). To determine whether baseline patient activation scores influenced participation and retention of participants in this study, a sensitivity analysis was performed, which showed that baseline activation scores for patients from CALD backgrounds who did not complete 12 months follow‐up (51.0 ± 13.8) and those who did (52.9 ± 20.1) were not different (Supporting Information: Table S1). Among patients from non‐CALD backgrounds, no difference was observed as well between those who did not complete 12 months follow‐up (58.4 ± 14.2) and those who did (58.6 ± 14.9). There were no differences with respect to other characteristics, except that patients from CALD backgrounds who did not complete 12 months follow‐up were older (71.8 ± 11.1) than those who did (66.0 ± 12.4) (Supporting Information: Table S1).

## 5 DISCUSSION

We studied the relationship between patient activation and CALD status among patients with diabetes and CKD and showed that patients from CALD backgrounds had lower activation scores compared to those from non‐CALD backgrounds at baseline. Additionally, we have shown that a codesigned integrated kidney and diabetes model of care significantly improved activation levels of patients from CALD backgrounds, but not those from non‐CALD backgrounds at 12 months follow‐up.

Patient activation is low among patients with diabetes and CKD as described previously,^13^, ^14^ but in the current study, we report even much lower activation scores among the subgroup of patients from CALD backgrounds. In our sample, patients from CALD backgrounds scored 7 points less on the PAM compared to those from non‐CALD backgrounds. Considering that a 1‐point incremental change in activation equates to an improvement in health outcomes of about 3% and a reduction in health costs of about 3%,^38^ it is clear that patients from CALD backgrounds may experience disparity in the quality and safety of health care.

Our finding of an association between low activation and CALD background is important in several ways. First, our study opens up an opportunity to explore potential causal and mediating factors for this relationship. A previous study among elderly minority patients^39^ reported that the relationship between African American race and lower patient activation was fully mediated by health literacy. Another study among patients who attended primary care clinics reported lower activation scores among Black participants but suggested that the relationship between race and activation could be mediated by income.^40^ Unfortunately, in our study, we did not collect health literacy and income data to confirm this finding. Second, when interpreting our results, it is important to consider that even though the PAM has been used across multiple racial, ethnic and language groups,^41^ it is unknown if the contextual components of PAM are race or culturally specific.^17^ Participants who speak English have been reported to score significantly higher than non‐English speakers^42^ and this may explain why patients from non‐CALD backgrounds scored higher in our study. Third, these results can inform the design of interventions that target people from CALD backgrounds among patients with comorbid diabetes and CKD to ensure disparities that exist in patient activation are identified and addressed.

One such intervention is our codesigned integrated kidney and diabetes model of care that significantly improved activation levels of patients from CALD backgrounds after 12 months. We observed an improvement of 8 points in patient activation, which doubled the minimal clinically important difference of 4 points^35^, ^43^ on the PAM scale. Components of this model of care have been described previously,^26^ but key features that may have led to improvements in patient activation among patients from CALD backgrounds include the focus on person‐centred care, interdisciplinary management of each patient and effective communication with the patient's primary care home team. With this approach, our model of care effectively improved access to health care by reducing barriers associated with navigating the healthcare system, for example, some primary care professionals who refer patients to the DKS felt that referrals for new patients were triaged and processed in a timely way compared to other individual specialist services.^44^ Additionally, the model of care allows for a strong and trusting relationship between healthcare professionals and patients to be built and this was facilitated by the ability of patients to see the same cohort of healthcare providers consistently.^45^ Supportive and trustworthy interactions enable patients to take a more active role in their health as they remove the inherent imbalance in power between patients and healthcare professionals.^46^ The improvement in patient activation is consistent with an Australian study^3^ that incorporated core principles of the Patient‐Centred Medical Home model among primary care patients presenting with chronic diseases.

We observed a significant improvement in activation among patients from CALD backgrounds even though both groups received the same intervention due to several reasons. First, patients with low activation at baseline are likely to benefit most since interventions designed to improve activation are usually tailored towards those with low levels of activation at baseline.^47^, ^48^, ^49^, ^50^ Second, patients from non‐CALD background who reported better activation scores at baseline may not have responded to the intervention because they had already attained optimal activation levels. In support of this, a prospective longitudinal study among patients with type 2 diabetes reported that patients who were at activation Level 3 at baseline were most likely to remain in Level 3 at follow‐up.^51^

This study has several strengths and limitations. The study did not only demonstrate that patient activation is low among patients with diabetes and CKD from CALD backgrounds, but that a codesigned integrated kidney and diabetes model of care can effectively improve patient activation in this group of patients who are largely underrepresented in research. In terms of limitations, our study was not originally designed to examine small subgroups of patients; however, at baseline, there were similarities with respect to demographic and clinical characteristics between patients from CALD and those from non‐CALD backgrounds. This meant that differences in activation scores at 12 months follow‐up could be associated with the intervention. Additionally, we had a good representation of patients from CALD backgrounds based on similarities of the proportion of patients who spoke a language other than English reported by the 2021 Australian census (21.1%)^52^ and in our study (27%). While it would have been interesting to determine the mediating effect of health literacy, we did not collect data to support these analyses. Future studies should examine whether health literacy has any mediating role in the relationship between patient activation and CALD status in patients with diabetes and CKD, and if this is the case, interventions that include elements targeted at improving health literacy and removing complexity from health systems need to be prioritised.

In conclusion, among patients with diabetes and CKD, those from CALD backgrounds report worse activation scores. Interventions that target people from CALD backgrounds among patients with comorbid diabetes and CKD, such as the integrated kidney and diabetes model of care, are needed to address disparities that exist in patient activation.

## AUTHOR CONTRIBUTIONS

*Study conception and design*: Edward Zimbudzi, Clement Lo, Sanjeeva Ranasinha, Tim Usherwood, Kevan R. Polkinghorne, Gregory Fulcher, Martin Gallagher, Stephen Jan, Alan Cass, Rowan Walker, Grant Russell, Greg Johnson, Peter G. Kerr, Sophia Zoungas. *Data curation*: Edward Zimbudzi, Kevan R. Polkinghorne, Sanjeeva Ranasinha. *Formal analysis*: Edward Zimbudzi, Kevan R. Polkinghorne. *Funding acquisition*: Clement Lo, Sophia Zoungas. *Investigation*: Edward Zimbudzi. *Methodology*: Edward Zimbudzi, Kevan R. Polkinghorne, Sophia Zoungas. *Project administration*: Edward Zimbudzi, Clement Lo, Sophia Zoungas. *Resources*: Sophia Zoungas. *Software*: Edward Zimbudzi. *Supervision*: Clement Lo, Sophia Zoungas, Peter G. Kerr. *Validation*: Sanjeeva Ranasinha. *Writing original draft*: Edward Zimbudzi, Kevan R. Polkinghorne, Sophia Zoungas. *Writing review and editing*: Edward Zimbudzi, Clement Lo, Sanjeeva Ranasinha, Tim Usherwood, Kevan R. Polkinghorne, Gregory Fulcher, Martin Gallagher, Stephen Jan, Alan Cass, Rowan Walker, Grant Russell, Greg Johnson, Peter G. Kerr, Sophia Zoungas. All authors have read and agreed to the published version of the manuscript.

## CONFLICT OF INTEREST STATEMENT

The authors declare no conflict of interest.

## Supporting information

Supporting information.

Supporting information.

Supporting information.

## Data Availability

Data are available on request from the authors.

## References

[1] Hibbard JH , Stockard J , Mahoney ER , Tusler M . Development of the patient activation measure (PAM): conceptualizing and measuring activation in patients and consumers: development of the patient activation measure (PAM). Health Serv Res. 2004;39(4 Pt 1):1005‐1026. 15230939 10.1111/j.1475-6773.2004.00269.x PMC1361049

[2] Yao F , Zheng M , Wang X , et al. Patient activation level and its associated factors in adults with chronic pain: a cross‐sectional survey. Medicine. 2021;100(19):e25929. 34106661 10.1097/MD.0000000000025929 PMC8133271

[3] John JR , Tannous WK , Jones A . Outcomes of a 12‐month patient‐centred medical home model in improving patient activation and self‐management behaviours among primary care patients presenting with chronic diseases in Sydney, Australia: a before‐and‐after study. BMC Fam Pract. 2020;21(1):158. 32770944 10.1186/s12875-020-01230-w PMC7414685

[4] Overbeek A , Rietjens JAC , Jabbarian LJ , et al. Low patient activation levels in frail older adults: a cross‐sectional study. BMC Geriatr. 2018;18(1):7. 29304752 10.1186/s12877-017-0696-9 PMC5756388

[5] Lubetkin EI , Lu WH , Gold MR . Levels and correlates of patient activation in health center settings: building strategies for improving health outcomes. J Health Care Poor Underserved. 2010;21:796‐808. 20693726 10.1353/hpu.0.0350

[6] Kim JY , Wineinger NE , Steinhubl SR . The influence of wireless self‐monitoring program on the relationship between patient activation and health behaviors, medication adherence, and blood pressure levels in hypertensive patients: a substudy of a randomized controlled trial. J Med Internet Res. 2016;18(6):e116. 27334418 10.2196/jmir.5429 PMC4935792

[7] Paukkonen L , Oikarinen A , Kähkönen O , Kaakinen P . Patient activation for self‐management among adult patients with multimorbidity in primary healthcare settings. Health Sci Rep. 2022;5(4):e735. 35873391 10.1002/hsr2.735 PMC9297377

[8] Greene J , Hibbard JH . Why does patient activation matter? An examination of the relationships between patient activation and health‐related outcomes. J Gen Intern Med. 2012;27(5):520‐526. 22127797 10.1007/s11606-011-1931-2 PMC3326094

[9] Kinney RL , Lemon SC , Person SD , Pagoto SL , Saczynski JS . The association between patient activation and medication adherence, hospitalization, and emergency room utilization in patients with chronic illnesses: a systematic review. Patient Educ Couns. 2015;98(5):545‐552. 25744281 10.1016/j.pec.2015.02.005

[10] Dumitra T , Ganescu O , Hu R , et al. Association between patient activation and health care utilization after thoracic and abdominal surgery. JAMA Surg. 2021;156(1):205002. 10.1001/jamasurg.2020.5002 PMC7643038 33146682

[11] Greene J , Hibbard JH , Sacks R , Overton V , Parrotta CD . When patient activation levels change, health outcomes and costs change, too. Health Aff. 2015;34(3):431‐437. 10.1377/hlthaff.2014.0452 25732493

[12] Blakemore A , Hann M , Howells K , et al. Patient activation in older people with long‐term conditions and multimorbidity: correlates and change in a cohort study in the United Kingdom. BMC Health Serv Res. 2016;16(1):582. 27756341 10.1186/s12913-016-1843-2 PMC5069882

[13] Zimbudzi E , Lo C , Ranasinha S , et al. Factors associated with patient activation in an Australian population with comorbid diabetes and chronic kidney disease: a cross‐sectional study. BMJ Open. 2017;7(10):e017695. 10.1136/bmjopen-2017-017695 PMC5665291 29061622

[14] Zimbudzi E , Lo C , Ranasinha S , et al. The association between patient activation and self‐care practices: a cross‐sectional study of an Australian population with comorbid diabetes and chronic kidney disease. Health Expect. 2017;20(6):1375‐13784. 28675539 10.1111/hex.12577 PMC5689227

[15] Khatri RB , Assefa Y . Access to health services among culturally and linguistically diverse populations in the Australian universal health care system: issues and challenges. BMC Public Health. 2022;22(1):880. 35505307 10.1186/s12889-022-13256-z PMC9063872

[16] Renzaho A , Renzaho C , Polonsky M . Left out, left off, left over: why migrants from non‐English speaking backgrounds are not adequately recognised in health promotion policy and programs. Health Promot J Austr. 2012;23(2):84‐85. 23088482 10.1071/he12084

[17] Charlot M , Winter MR , Cabral H , et al. Patient activation mediates health literacy associated with hospital utilization among Whites. Health Lit Res Pract. 2017;1(3):e128‐e135. 29806046 10.3928/24748307-20170621-01 PMC5967226

[18] Coulter A , Entwistle VA , Eccles A , Ryan S , Shepperd S , Perera R . Personalised care planning for adults with chronic or long‐term health conditions. Cochrane Database Syst Rev. 2015;2015(3):CD010523. 25733495 10.1002/14651858.CD010523.pub2 PMC6486144

[19] Reuben DB , Tinetti ME . Goal‐oriented patient care—an alternative health outcomes paradigm. N Engl J Med. 2012;366(9):777‐779. 22375966 10.1056/NEJMp1113631

[20] Baxter S , Johnson M , Chambers D , Sutton A , Goyder E , Booth A . The effects of integrated care: a systematic review of UK and international evidence. BMC Health Serv Res. 2018;18(1):350. 29747651 10.1186/s12913-018-3161-3 PMC5946491

[21] Rocks S , Berntson D , Gil‐Salmerón A , et al. Cost and effects of integrated care: a systematic literature review and meta‐analysis. Eur J Health Econ. 2020;21(8):1211‐1221. 32632820 10.1007/s10198-020-01217-5 PMC7561551

[22] Smith SG , Pandit A , Rush SR , Wolf MS , Simon CJ . The role of patient activation in preferences for shared decision making: results from a national survey of U.S. adults. J Health Commun. 2016;21(1):67‐75. 26313690 10.1080/10810730.2015.1033115 PMC4706032

[23] Nguyen LH , Anyane‐Yeboa A , Klaser K , et al. The mental health burden of racial and ethnic minorities during the COVID‐19 pandemic. PLoS One. 2022;17(8):e0271661. 35947543 10.1371/journal.pone.0271661 PMC9365178

[24] Gainsburg I , Derricks V , Shields C , et al. Patient activation reduces effects of implicit bias on doctor‐patient interactions. Proc Natl Acad Sci USA. 2022;119(32):e2203915119. 35914161 10.1073/pnas.2203915119 PMC9371681

[25] Hall WJ , Chapman MV , Lee KM , et al. Implicit racial/ethnic bias among health care professionals and its influence on health care outcomes: a systematic review. Am J Public Health. 2015;105(12):e60‐e76. 10.2105/AJPH.2015.302903 PMC4638275 26469668

[26] Lo C , Zimbudzi E , Teede H , et al. An Australian model of care for co‐morbid diabetes and chronic kidney disease. Nephrology. 2018;23(8):711‐717. 29405503 10.1111/nep.13232

[27] Zimbudzi E , Lo C , Ranasinha S , et al. Health‐related quality of life among patients with comorbid diabetes and kidney disease attending a codesigned integrated model of care: a longitudinal study. BMJ Open Diabetes Res Care. 2020;8(1):e000842. 10.1136/bmjdrc-2019-000842 PMC6954749 31958294

[28] Zimbudzi E , Lo C , Ranasinha S , et al. A co‐designed integrated kidney and diabetes model of care improves mortality, glycaemic control and self‐care. Nephrol Dial Transplant. 2022;37(8):1472‐1481. 34314493 10.1093/ndt/gfab230

[29] Levey AS , Stevens LA , Schmid CH , et al. A new equation to estimate glomerular filtration rate. Ann Intern Med. 2009;150(9):604‐612. 19414839 10.7326/0003-4819-150-9-200905050-00006 PMC2763564

[30] Von Elm E , Altman DG , Egger M , Pocock SJ , Gotzsche PC , Vandenbroucke JP . The Strengthening the Reporting of Observational Studies in Epidemiology (STROBE) statement: guidelines for reporting observational studies. Lancet. 2007;370(9596):1453‐1457. 18064739 10.1016/S0140-6736(07)61602-X

[31] Lo C , Teede H , Fulcher G , et al. Gaps and barriers in health‐care provision for co‐morbid diabetes and chronic kidney disease: a cross‐sectional study. BMC Nephrol. 2017;18(1):80. 28245800 10.1186/s12882-017-0493-x PMC5331625

[32] Australian Bureau of Statistics . *Standards for Statistics on Cultural and Language Diversity. Standards for Statistics on Cultural and Language Diversity*. 1999.

[33] Pham TTL , Berecki‐Gisolf J , Clapperton A , O'Brien KS , Liu S , Gibson K . Definitions of culturally and linguistically diverse (CALD): a literature review of epidemiological research in Australia. Int J Environ Res Public Health. 2021;18(2):737. 33467144 10.3390/ijerph18020737 PMC7830035

[34] Sawrikar P , Katz I . Enhancing Family and Relationship Service Accessibility and Delivery to Culturally and Linguistically Diverse Families in Australia. Australian Institute of Family Studies; 2008.

[35] Hibbard JH , Mahoney ER , Stockard J , Tusler M . Development and testing of a short form of the patient activation measure. Health Serv Res. 2005;40:1918‐1930. 16336556 10.1111/j.1475-6773.2005.00438.x PMC1361231

[36] InsigniaHealth . *Patient Activation Measure (PAM) 13TM License Materials*. 2010.

[37] Zimbudzi E , Lo C , Ranasinha S , et al. Patient reported barriers are associated with low physical and mental well‐being in patients with co‐morbid diabetes and chronic kidney disease. Health Qual Life Outcomes. 2018;16(1):215. 30454062 10.1186/s12955-018-1044-2 PMC6245917

[38] Janamian T , Greco M , Cosgriff D , Baker L , Dawda P . Activating people to partner in health and self‐care: use of the patient activation measure. Med J Aust. 2022;216(suppl 10):S5‐S8. 35665937 10.5694/mja2.51535 PMC9328281

[39] Gwynn KB , Winter MR , Cabral HJ , et al. Racial disparities in patient activation: evaluating the mediating role of health literacy with path analyses. Patient Educ Couns. 2016;99(6):1033‐1037. 26809936 10.1016/j.pec.2015.12.020 PMC4912873

[40] Holt JM , Winn A , Cusatis R , Talsma A , Crotty BH . Racial disparities in patient activation: the role of economic diversity. West J Nurs Res. 2021;43(6):517‐529. 33012264 10.1177/0193945920963130

[41] Alexander J , Hearld L , Mittler JN . Patient–physician role relationships and patient activation: the moderating effects of race and ethnicity. Med Care Res Rev. 2014;71(5):472‐495. 25027408 10.1177/1077558714541967

[42] Lubetkin EI , Zabor EC , Brennessel D , Kemeny MM , Hay JL . Beyond demographics: differences in patient activation across new immigrant, diverse language subgroups. J Community Health. 2014;39:40‐49. 23918645 10.1007/s10900-013-9738-1 PMC3947232

[43] Hibbard JH , Greene J , Tusler M . Improving the outcomes of disease management by tailoring care to the patient's level of activation. Am J Manag Care. 2009;15(6):353‐360. 19514801

[44] Zimbudzi E , Lo C , Robinson T , et al. The impact of an integrated diabetes and kidney service on patients, primary and specialist health professionals in Australia: a qualitative study. PLoS One. 2019;14(7):e0219685. 31306453 10.1371/journal.pone.0219685 PMC6629146

[45] Aseltine Jr. RH , Sabina A , Barclay G , Graham G . Variation in patient‐provider communication by patient's race and ethnicity, provider type, and continuity in and site of care: an analysis of data from the Connecticut Health Care Survey. SAGE Open Med. 2016;4:205031211562516. 10.1177/2050312115625162 PMC4724761 26835017

[46] Becker ER , Roblin DW . Translating primary care practice climate into patient activation: the role of patient trust in physician. Med Care. 2008;46:795‐805. 18665059 10.1097/MLR.0b013e31817919c0

[47] Ryvicker M , Feldman PH , Chiu YL , Gerber LM . The role of patient activation in improving blood pressure outcomes in Black patients receiving home care. Med Care Res Rev. 2013;70(6):636‐652. 23864112 10.1177/1077558713495452

[48] Carroll JK , Tobin JN , Luque A , et al. “Get Ready and Empowered About Treatment” (GREAT) study: a pragmatic randomized controlled trial of activation in persons living with HIV. J Gen Intern Med. 2019;34(9):1782‐1789. 31240605 10.1007/s11606-019-05102-7 PMC6712153

[49] Westland H , Schuurmans MJ , Bos‐Touwen ID , et al. Effectiveness of the nurse‐led activate intervention in patients at risk of cardiovascular disease in primary care: a cluster‐randomised controlled trial. Eur J Cardiovasc Nurs. 2020;19(8):721‐731. 32375491 10.1177/1474515120919547 PMC7817988

[50] Hibbard JH , Greene J . What the evidence shows about patient activation: better health outcomes and care experiences; fewer data on costs. Health Aff. 2013;32(2):207‐214. 10.1377/hlthaff.2012.1061 23381511

[51] Regeer H , van Empelen P , Bilo HJG , de Koning EJP , Huisman SD . Change is possible: how increased patient activation is associated with favorable changes in well‐being, self‐management and health outcomes among people with type 2 diabetes mellitus: a prospective longitudinal study. Patient Educ Couns. 2022;105(4):821‐827. 34274165 10.1016/j.pec.2021.07.014

[52] Australian Bureau of Statistics . *Language Used at Home (LANP)*. 2021.

