# Supplementary material for: A codesigned integrated kidney and diabetes model of care improves patient activation among patients from culturally and linguistically diverse backgrounds
Source: Health Expect. 2023 Aug 27;26(6):2584–93. doi: 10.1111/hex.13859 (PMC10632627; doi:10.1111/hex.13859)
Supplement: Supplementary file 1 — Supporting information. [file HEX-26--s001.pdf]

DRP: Diabetes Renal Project
(Doctors Survey - Health Indicators)

Thank-you for participating in this large multi-centre research project, called the Diabetes Renal Project (DRP). This National Health and Medical Research Council (NHMRC) partnership project is being conducted by Monash University, in partnership with Monash Health, Alfred Health, Royal North Shore Hospital, Concord Repatriation General Hospital, The George Institute for Global Health, Diabetes Australia, and Kidney Health Australia.

INSTRUCTIONS

PLEASE:

Use a black **BIRO**, (DO NOT use a pencil or a fountain or felt tip pen)

Please **PRINT** in **CAPITAL** letters and stay within the box provided for text.

If you make a **mistake when writing**, cross it out with one thick line and write your correct answer above the box.

To answer a multiple choice question place a **CROSS INSIDE** the box like this: ☒

If you make a **mistake**, place a diagonal line through the incorrect answer like this: ☒ and then put a cross in the box of your preferred response.

Write dates using leading zeros (e.g. **6th April 2011 = 06/04/2011**)

DO NOT USE liquid paper to correct mistakes.

AVOID folding the form.

Please complete every page of the questionnaire. Sometimes questions may seem very similar or repetitious but they are all a little different, so please answer each question.

THANK YOU

Hospital ID: ☐ Site Staff ID: ☐☐☐☐☐ Participant ID: ☐☐☐☐

Date ☐☐ / ☐☐ / ☐☐☐☐
day month year

Health Indicators (Doctors Survey)

Section 1: Demographic of Patient Participant

1. Age *(years)* ☐☐

2. Gender ☐ Male ☐ Female

3. Participant Post-code ☐☐☐☐

4. Aboriginal background ☐ No ☐ Yes

5.Torres Strait Islander background ☐ No ☐ Yes

6. Maori/Pacific Strait Islander background ☐ No ☐ Yes

7. Is the participant a current smoker ?

☐ No → Skip to Q 8

☐ Yes → 7.1. **Average number of cigarettes smoked per day?** ☐☐

8. Has the participant previously smoked ?

☐ No → Skip to Q 9

☐ Yes → 8.1. **Average number of cigarettes smoked per day?** ☐☐

9. Does the participant currently drink alcohol?

☐ No → Skip to Q 10

☐ Yes → 9.1. **Average number of standard drinks per week?** ☐☐

Section 2: Examination Findings

Please complete with the most recent examination findings and date of examination

10. Blood Pressure - *(the average of 3 readings measured after 5 minutes sitting)*

☐☐☐ / ☐☐☐ mmHg → **10.1** ☐☐ / ☐☐ / ☐☐☐☐
day month year

11. Heart Rate ☐☐☐ Bpm → **11.1** ☐☐ / ☐☐ / ☐☐☐☐
day month year

12. Weight ☐☐☐.☐ Kg → **12.1** ☐☐ / ☐☐ / ☐☐☐☐
day month year

13. Height ☐.☐☐ Metres → **13.1** ☐☐ / ☐☐ / ☐☐☐☐
day month year

At the most recent examination, does the participant have the following conditions:

14a. New loss of vibratory sensation (both feet)

☐ No ☐ Yes → Date of examination **14a.1** ☐☐ / ☐☐ / ☐☐☐☐
day month year

☐ Not examined/unknown

14b. New loss of ankle reflexes (both legs)

☐ No ☐ Yes → Date of examination **14b.1** ☐☐ / ☐☐ / ☐☐☐☐
day month year

☐ Not examined/unknown

14c. New loss of light touch (eg. loss of pressure sensation with 10gm force monofilament)

☐ No ☐ Yes → Date of examination **14c.1** ☐☐ / ☐☐ / ☐☐☐☐
day month year

☐ Not examined/unknown

Section 2: Examination Findings (cont)

15. Foot ulcers

☐ No ☐ Yes → Date of examination **15.1** ☐☐ / ☐☐ / ☐☐☐☐ (day / month / year)

☐ Not examined/unknown

16. Foot deformity

☐ No ☐ Yes → Date of examination **16.1** ☐☐ / ☐☐ / ☐☐☐☐ (day / month / year)

☐ Not examined/unknown

Section 3: Medical History

17. Diabetes Type ☐ Type 1 ☐ Type 2

18. Duration of diabetes ☐☐ *years* ☐☐ *months*

OR ☐ Unknown/not documented

Has the participant experienced any of the following complications/comorbidities?

**19. Ischemic Heart Disease?**	☐ No	☐ Yes
**20. Stroke?**	☐ No	☐ Yes
**21. Peripheral Vascular disease?**	☐ No	☐ Yes
**22. Diabetic Retinopathy?**	☐ No	☐ Yes
**23. Peripheral Neuropathy?**	☐ No	☐ Yes
**24. Diabetic Nephropathy?**	☐ No	☐ Yes
**25. Hypertension**	☐ No	☐ Yes
**26. Dyslipidemia**	☐ No	☐ Yes

27. Does the participant have a family history of heart disease? ☐ No ☐ Yes

OR ☐ Unknown/not documented

---

28. Duration of nephrological care ☐☐ *years* ☐☐ *months* **OR** ☐ Unknown/not documented

29. Kidney disease stage *(select one option)* ☐ Stage 3a ☐ Stage 3b ☐ Stage 4 ☐ Stage 5

30. Is the patient currently on dialysis?

☐ No → Skip to Q 31

☐ Yes → **30.1** **Haemodialysis** ☐ No ☐ Yes → **30.2** Number of months on dialysis ☐☐☐

30.3 **Peritoneal** ☐ No ☐ Yes → **30.4** Number of months on dialysis ☐☐☐

Section 3: Medical History (cont)

31. Prior to their current dialysis, has the patient been on any other form of dialysis?

☐ No → Skip to Q 32

☐ Yes→ **31.1 Haemodialysis?**

☐ No ☐ Yes

Date commenced **31.2** ☐☐ / ☐☐ / ☐☐☐☐ (day / month / year)

Date ceased **31.3** ☐☐ / ☐☐ / ☐☐☐☐ (day / month / year)

31.4 Peritoneal dialysis?

☐ No ☐ Yes

Date commenced **31.5** ☐☐ / ☐☐ / ☐☐☐☐ (day / month / year)

Date ceased **31.6** ☐☐ / ☐☐ / ☐☐☐☐ (day / month / year)

32. Has the patient had a kidney transplant?

☐ No → Skip to Q 33

☐ Yes→ **32.1 Date of transplant** ☐☐ / ☐☐ / ☐☐☐☐ (day / month / year)

OR ☐ Unknown/not documented

Section 4: Medical Care of Diabetes and Chronic Kidney Disease

33. How often does the participant monitor his/her diabetes with a blood glucose monitor? *(select one option)*

☐ ≥ 3 times per day ☐ Once per day (daily) ☐ Once per week (weekly) ☐ Uncertain

☐ 2 times per day ☐ A few times per week ☐ Rarely ☐ Not documented

34. Please indicate when the participant was last referred/seen by the following health professionals.
(Select the appropriate response for each health professional).

	Not referred/reviewed by this health professional	3 months or less	4-12 months ago	13-24 months ago	As required	Uncertain
**a.** Endocrinologist	☐	☐	☐	☐	☐	☐
**b.** Nephrologist	☐	☐	☐	☐	☐	☐
**c.** Diabetes Nurse Educator	☐	☐	☐	☐	☐	☐
**d.** Renal Nurse Practitioner	☐	☐	☐	☐	☐	☐
**e.** Optometrist	☐	☐	☐	☐	☐	☐
**f.** Ophthalmologist	☐	☐	☐	☐	☐	☐
**g.** Podiatrist	☐	☐	☐	☐	☐	☐
**h.** Dentist	☐	☐	☐	☐	☐	☐
**i.** Dietician	☐	☐	☐	☐	☐	☐
**j.** Social Worker	☐	☐	☐	☐	☐	☐

27311

Hospital ID: [ ] Site Staff ID: [ ][ ][ ][ ][ ] Participant ID: [ ][ ][ ][ ]

Section 5: Medications

35. Is the participant on Insulin?

☐ No → Skip to Q 36

☐ Yes→ **35.1 Is the participant on an Insulin pump?** ☐ No ☐ Yes

35.2 What type of insulin? *(select all that apply)*

☐ Long acting ☐ Short acting ☐ Rapid acting ☐ Basal

36. Is the participant on diabetes tablets?

☐ No → Skip to Q 34

☐ Yes→ **Does the participant take:**

**36.1 Metformin?**	☐ No	☐ Yes
**36.2 Sulphonylurea?**	☐ No	☐ Yes
**36.3 Glitazone?**	☐ No	☐ Yes
**36.4 Acarbose?**	☐ No	☐ Yes
**36.5 Gliptin (DPP4 inhibitor)?**	☐ No	☐ Yes
**36.6 GLP1 agonist?** **(e.g exenatide or liraglutide)**	☐ No	☐ Yes
**36.7 SGLT2 inhibitors?**	☐ No	☐ Yes

37. Other medications - is the participant taking:

**37.1 ACE inhibitor?**	☐ No	☐ Yes
**37.2 Angiotensin2 Receptor Blocker?**	☐ No	☐ Yes
**37.3 Other Antihypertensives?**	☐ No	☐ Yes
**37.4 Statin?**	☐ No	☐ Yes
**37.5Fibrate?**	☐ No	☐ Yes
**37.6 Erythropoieting Stimulating Agent?**	☐ No	☐ Yes
**37.7 Phosphate binder?**	☐ No	☐ Yes
**37.8 Iron Supplementation (IV or Oral)?**	☐ No	☐ Yes

36.8 Other diabetes medication *(please list below)*

[ ]

27311

Hospital ID: ☐ Site Staff ID: ☐☐☐☐☐ Participant ID: ☐☐☐☐

Section 6: Investigations

38. Has a HbA1c test been performed in the last 3 months? ☐ No ☐ Yes

Please record the most recent HbA1c result

38.1 HbA1c ☐☐☐ mmol/mol ***and*** **38.2** ☐☐.☐ % → **38.3** Date of test ☐☐ / ☐☐ / ☐☐☐☐ (day / month / year)

39. Please enter details below of the most recent lipid profile results:

39.1 Total Cholesterol ☐☐.☐ mmol/L

39.2 LDL Cholesterol ☐.☐☐ mmol/L

39.3 HDL Cholesterol ☐☐.☐☐ mmol/L

39.4 Triglycerides ☐.☐☐ mmol/L

39.5 Date of test ☐☐ / ☐☐ / ☐☐☐☐ (day / month / year)

OR ☐ Not tested

40. Please enter details below of the most recent serum biochemistry profile results:

40.1 Potassium ☐.☐ mmol/L

40.2 Creatinine ☐☐☐☐ µmol/L

40.3 Calcium ☐.☐☐ mmol/L

40.4 Phosphate ☐.☐☐ mmol/L

40.5 Parathyroid hormone (PTH) ☐☐☐.☐
(result within last 6 months)

40.5.1 Units ☐ pmol/L ☐ ng/L

OR ☐ Not done within the past 6 months

40.6 eGFR ☐☐☐ mL/min per $1.73m^2$

40.7 Albumin ☐☐☐ g/L

40.8 Date of test ☐☐ / ☐☐ / ☐☐☐☐ (day / month / year)

(For PTH, please record result from within the past 6 months of this date)

OR ☐ Not tested

41. Please record the most recent spot urine albumin / creatinine ratio (ACR):

☐☐☐☐.☐☐ mg/mmol **40.1 Date of test** ☐☐ / ☐☐ / ☐☐☐☐ (day / month / year) **OR** ☐ Not tested

42. If you have used another method to measure microalbumin / proteinuria please record details below:

☐☐☐☐.☐☐ **42.1 Units** ☐ mg/L ☐ mg/24hr ☐ µg/min ☐ g/mmol ☐ g/L

42.2 Date of test ☐☐ / ☐☐ / ☐☐☐☐ (day / month / year) **OR** ☐ Not tested

43. Please enter the most recent Haemoglobin test result: ☐☐☐ g/L **43.1 Date of test** ☐☐ / ☐☐ / ☐☐☐☐ (day / month / year)

OR ☐ Not tested
