# Supplementary material for: A codesigned integrated kidney and diabetes model of care improves patient activation among patients from culturally and linguistically diverse backgrounds
Source: Health Expect. 2023 Aug 27;26(6):2584–93. doi: 10.1111/hex.13859 (PMC10632627; doi:10.1111/hex.13859)
Supplement: Supplementary file 2 — Supporting information. [file HEX-26--s002.docx]

The caption is **DRP: Diabetes Renal Project (Doctors Survey - Health Indicators)**.
