# Supplementary material for: A codesigned integrated kidney and diabetes model of care improves patient activation among patients from culturally and linguistically diverse backgrounds
Source: Health Expect. 2023 Aug 27;26(6):2584–93. doi: 10.1111/hex.13859 (PMC10632627; doi:10.1111/hex.13859)
Supplement: Supplementary file 3 — Supporting information. [file HEX-26--s003.docx]

**Supplementary Table 1**: Baseline characteristics of patients who completed and those who did not compete follow up

|  | **All patients at baseline** | **Completed**  **(all patients)** | **Completed**  **(non-CALD)** | **Did not complete**  **(non-CALD)** | **Completed**  **(CALD)** | **Did not complete**  **(CALD)** |
| --- | --- | --- | --- | --- | --- | --- |
| Participants (n) | 290 | 170 | 126 | 87 | 44 | 33 |
| Age (years) | 66.6 (11.9) | 66 (11.4) | 66 (11.1) | 65.9 (12.6) | 66.0 (12.4) | 71.8 (11.1) * |
| Gender, n (%) |  |  |  |  |  |  |
| Female | 106 (36.5) | 62 (36.5) | 45 (35.7) | 31 (35.6) | 17 (38.6) | 13 (39.4) |
| Male | 184 (63.5) | 108 (63.5) | 81 (64.3) | 56 (64.4) | 27 (61.4) | 20 (60.6) |
| Duration of diabetes (years) | 16.1 (8.9) | 16.5 (8.6) | 16.7 (8.8) | 15.3 (9.3) | 16.0 (8.1) | 16.2 (10.0) |
| Stages of CKD, n (%) |  |  |  |  |  |  |
| 3a | 56 (19.3) | 35 (20.6) | 22 (17.5) | 16 (18.4) | 13 (29.5) | 5 (15.2) |
| 3b | 109 (37.5) | 71 (41.8) | 56 (44.4) | 27 (31.0) | 15 (34.1) | 11 (33.3) |
| 4 | 68 (23.5) | 35 (20.6) | 23 (18.3) | 25 (28.8) | 12 (27.3) | 8 (24.2) |
| 5 (including dialysis) | 57 (19.7) | 29 (17.0) | 25 (19.8) | 19 (21.8) | 4 (9.1) | 9 (27.3) |
| Patient activation | 56.8 (15.6) | 57.1 (16.5) | 58.6 (14.9) | 58.4 (14.2) | 52.9 (20.1) | 51.0 (13.8) |

CALD, Culturally and linguistically diverse background; CKD, chronic kidney disease; eGFR; *p=0.04
